# Extremely high prevalence of antiseptic resistant Quaternary Ammonium Compound *E* gene among clinical isolates of multiple drug resistant *Acinetobacter baumannii* in Malaysia

**DOI:** 10.1186/s12941-015-0071-7

**Published:** 2015-03-11

**Authors:** Mohammad Reza Babaei, Anita Sulong, Rukman Awang Hamat, Syafinaz Amin Nordin, Vasantha Kumari Neela

**Affiliations:** Department of Medical Microbiology and Parasitology, Faculty of Medicine and Health Sciences, Universiti Putra Malaysia, Serdang, 43400 Malaysia; Department of Medical Microbiology and Immunology, Faculty of Medicine, Universiti Kebangsaan Malaysia Medical Centre, Bandar Tun Razak, Cheras, Kuala Lumpur, 56000 Malaysia

**Keywords:** Antiseptic, *Acinetobacter baumannii*, Multiple drug resistance

## Abstract

**Background:**

Antiseptics are commonly used for the management of MDR (multiple drug resistance) pathogens in hospitals. They play crucial roles in the infection control practices. Antiseptics are often used for skin antisepsis, gauze dressing, preparation of anatomical sites for surgical procedure, hand sterilization before in contact with an infected person, before an invasive procedure and as surgical scrub.

**Methods:**

We screened 122 multiple drug resistant *Acinetobacter baumannii* (MDRAB) isolated from admitted patients in one of the tertiary care hospital in Malaysia for the presence of antiseptic resistant genes *qacA* and *qacE* (Quaternary Ammonium Compound) and susceptibility towards chlorhexidine (CLX), benzalkonium (BZK) and benzethonium (BZT).

**Results:**

Eighty-nine (73%) isolates harboured *qacE* gene, while none were positive for *qacA*. The MIC ranged from 0.2 to 0.6 for CLX, 0.02 to 0.2 for BZK and 0.04 to 0.2 μg/mL for BZT. The highest number of *qacE* positive isolates were obtained from surgery (n = 24; 27%; p < 0.05), followed by medical ward (n = 23; 25.8%) and ICU (n = 21; 23.6%). Majority of the isolates from wound swabs (n = 33; 37%), T/aspirate (n = 16; 18%) and tissue (n = 10; 11.2%) harboured the *qacE* genes.

**Conclusion:**

The present investigation showed high prevalence of *qacE* gene among the studied isolates. Antiseptics are important components of infection control, continuous monitoring of antiseptics use in the hospital is cautioned.

## Background

Multiple drug resistance *Acinetobacter baumannii* (MDRAB) ranks top among the nosocomial pathogen due to their environmental elasticity, ability to colonize various body sites of hospitalized patients, long-time persistence, association with multiple drug resistance and their successful outbreak potential [1-5]. It causes a wide spectrum of nosocomial infections which includes infections of bloodstream, urinary and respiratory tract, and ventilator associated pneumonia commonly among Intensive Care Unit patients (ICU) [6,7].

The main sources of transmission implicated with *A. baumannii* infections are person to person contact or through contaminated surface [8] and previous room occupancy by patients with *A. baumannii* infection or colonization. Epidemiological studies have clearly shown that hospital environment and colonized patients as the major reservoirs of *A. baumannii* infections [9]. Management of *A. baumannii* infections is the greatest challenge for patients, clinicians and infection control physicians. Infection control interventions such as patient screening, cohort isolation, hand hygiene compliance, surveillance of environmental contamination, enhanced cleaning and environmental disinfection have been shown to reduce nosocomial infection rates and outbreaks due to *A. baumannii* in various studies [4,10,11].

Antiseptics are increasingly used in hospitals to control the dissemination of nosocomial infections. The regular use of antiseptics in hospital has raised concerns about its resistance. A very recent study by Suwantarat [12] has shown that bacteria causing life threatening infections in seriously ill patients are now becoming less susceptible to the commonly used antiseptics in the hospital. The study compared patients in ICU who received daily antiseptic washes with non ICU patients who did not receive any antiseptic baths. It was found that patients who received regular antiseptics baths showed less susceptibility to CLX compared to those who did not receive any antiseptic washes. Antiseptic resistance is encoded by the *qac* genes. To date several *qac* genes such as *qac A/B* genes [13-15], *qac C/D* also known as *smr* [15], *qacE/F* [16,17]*, qacG* [18], *qacH* [19,20], *qac J* [21,22] and *qac Z* [23,24] have been reported. *Qac A/B* followed by *qac C/D* genes is frequently associated with gram positive [25] and [26], while *qac E* is commonly seen in gram negative bacteria.

Several studies from Asia including Malaysia have shown continuous increase of highly multiple drug resistant *A. baumannii* in Asia [27-30]. A recent study [27] demonstrated correlation between reduced susceptibility to disinfectants and multidrug resistance among clinical isolates of *Acinetobacter* species. Although several studies have shown the prevalence of antibiotic resistance in *A. baumanni*, only very few reports are available on the antiseptic and disinfectant susceptibility. In Malaysia, despite, *A. baumannii* being one of the serious nosocomial pathogen, its susceptibility towards antiseptics and disinfectant is largely unknown. Therefore, in this study we investigated the antiseptic and disinfectant susceptibility and carriage of corresponding resistant genes in *A. baumannii* isolated from patients admitted in one of the tertiary care teaching hospital in Malaysia. The study was conducted in Universiti kebangsan Malaysia Medical Centre, as it is in the capital of the country, which receives population from all over Malaysia. The rising trends of *A. baumannii* infections in the hospital prompted to investigate the antiseptic and disinfectant susceptibility.

## Methods

### Bacterial isolates

A total of 122 non-repetitive multiple drug resistance *Acinetobacter baumannii* (MDRAB) isolates collected from various clinical specimens (5 from blood, 41 wound swabs, 26 tracheal aspirate, 14 urine, 14 tissue, 10 sputum, 12 from others) from February 2012 to January 2013 from a 900 bedded hospital UKMMC was investigated. The isolates were confirmed in the hospital by the standard microbiological methods and also by AP 20NE (bioMérieux, France). The isolates were reconfirmed as *A. baumannii* by standard methods (oxidase, catalase, TSI, MRVP, Simmon citrate, motility, urease and Gram staining) in our laboratory stationed at Department of Medical Microbiology and Parasitology, Faculty of Medicine and Health Sciences, Universiti Putra Malaysia. An isolate is defined as MDRAB when it is resistant to more than three classes of antibiotics tested by disc diffusion test.

### PCR assay for qacA and qacE

Total genomic DNA was extracted from MDRAB isolates using GF-1 Bacterial DNA Extraction Kit (Vivantis Technologies, Malaysia). All isolates were screened for the presence of *qacA* [31] (5′GCAGAAAGTGCAGAGTTCG-3′ and 5′ TCAACCGAATAGAGTGAACTTATCT-3′) and *qacE* [32] (*QacE* F: 5′-GCGAAGTAATCGCAACATCC-3′ and *Qac E* R: 5′ GCCCCATACCTACAAAGCC-3′) genes. Two representative isolates for each gene was sequenced (First BASE Laboratories, Malaysia) and used as positive control. The sequence of the genes were confirmed by blastN program in GenBank (http://www.ncbi.nlm.nih.gov).

### Antiseptic susceptibility

All antiseptics chlorhexidine (CLX), benzalkonium (BZK) and benzethonium (BZT) were purchased from Sigma Aldrich (St Louis, MO, USA). A stock of antiseptics containing 100 mg/L of CLX, BZK and BZT in deionized water was prepared and stored at 4°C. MICs of antiseptics were determined by the broth microdilution method according to the Clinical and Laboratory Standards Institute [1]. Since there was no standard breakpoints available for antiseptics against *A. baumannii*, we tested a 2 fold dilutions from 4% to 0.00006%. A standard bacterial concentration of McFarland Standard 0.5 (1.5 × 10^8^ CFU/mL) was used. Briefly 50 μl of bacterial suspension was added from well one to twelve in a 96 well plate. To well one, 50 μl of 4% antiseptics was added. Upon mixing well, 50 μl was transferred to next well and continued until last well. The dilutions included 4%, 2%, 1%, 0.5%, 0.25%, 0.125%, 0.0625%, 0.0312%, 0.0156%, 0.0078%, 0.0039% and 0.0019%. Susceptibility was interpreted based on the turbidity on the inoculum after incubation at 37°C for 24 hrs.

## Results

Among the 122 isolates tested, *qacE* was found to be present in 89 isolates (72.95%), none of the isolates carried *qacA* gene. In general for *qacA* positive isolates*,* the MIC ranged from 0.2 to 0.6 μg/mL for CLX, 0.02 to 0.2 μg/mL for BZK and 0.04 to 0.2 μg/mL for BZT. For *qacE* negative isolates, MIC ranged from 0.04 to 0.3 μg/mL for CLX, 0.01 for 0.08 μg/mL for BZK and 0.02 to 0.08 μg/mL for BZT. Highest *qacE* positive isolates were obtained from surgery (n = 24; 27%), followed by medical ward (n = 23; 25.8%) and ICU (n = 21; 23.6%) as illustrated in Tables 1 and 2. Isolation of *qacE* positive MDRAB was found to be significantly higher in the surgical ward (p < 0.05). Majority of the isolates from wound swabs (n = 33; 37%), T/aspirate (n = 16/18%) and tissue (n = 10/11.2%) harboured the *qacE* genes.

**Table 1:** **Isolation of** ***qacE*** **positive MDRAB from different wards**

**Ward**	**Number of isolates (%)**	
	***qacE*** **positive (n = 89)**	***P*** **value**
ICU	21 (23.6%)	>0.05
Neurosurgery	2 (2.2%)	>0.05
Orthopaedics	7 (7.9%)	>0.05
Urology	4 (4.5%)	>0.05
Surgery	24 (27%)	<0.05*
Medical	23 (25.8%)	>0.05
Burn	2 (2.3%)	>0.05
Others	6 (6.7%)	>0.05

<0.05*Indicates statistically significant.

**Table 2:** **Isolation of** ***qacE*** **positive MDRAB from different clinical specimens**

**Clinical specimens**	***qacE*** **positive (n = 89)**	***P*** **value**
Tracheal aspirate	16 (18%)	>0.05
Wound swabs	33 (37%)	>0.05
Tissue	10 (11.2%)	>0.05
Blood	4 (4.5%)	>0.05
Urine	13 (14.6%)	>0.05
Sputum	6 (6.8%)	>0.05
Others	7 (7.9%)	>0.05

## Discussion

MDRAB is the biggest challenge for the infection control unit in every hospital setting. Growing resistance to every licensed antimicrobial agent against MDRAB including carbapenems to date has made this organism of global concern. Resistance rates may likely increase the treatment failures and mortality rates [29,30]. Unfortunate acquisition of MDRAB that leads to life threatening blood stream infections and pneumonia in the hospital could be prevented if proper hygienic measures are implemented, practiced and monitored regularly.

Antiseptics are widely used for infection control. They are applied to living tissue to reduce the possibility of infection, sepsis, or putrefaction and especially to keep the environment and the inanimate objects clean (for e.g. ventilators, catheter) from microbial communities. In the studied hospital, we found majority of the MDRAB isolates 89 (73%) to harbour the *qacE* gene. However, the MIC for all the QAC’s tested was far less than the concentration used in the hospitals (0.5%–4% mL/liter_)_ [33]. A study [34] showed higher MIC for *S. aureus* that harboured *qacA/B* gene. In the current study for MDRAB, no significant difference (p > 0.05) was observed in MIC for *qacE* positive and negative isolates. The high carriage of *qacE* positive MDRAB in surgical ward (p < 0.05) is not surprising as it is known that antiseptics are very frequently used in surgical wards to reduce the skin and soft tissue infections and also to reduce the postsurgical length of stay and duration of antibiotic therapy [3]. Central venous (CV) catheters are commonly used in the ICU as it plays crucial role in the management of critically ill patients.

In the current study, we found that majority of MDRAB isolates from ICU harbour *qacE* gene. Although the use of antiseptic impregnated catheters reduces catheter related bloodstream infections, emergence of antiseptic resistant strains are still on the rise. A recent study [34] reported high rates of *qacA* and *qacB* positive MRSA isolation from chlorhexidine impregnated catheter related bloodstream infections.

Stable resistance for antiseptics and antibiotics could be obtained for MDRAB when they are exposed step-wise with gradually increasing concentrations of QAC’s as seen in *Pseudomonas aeuroginosa* and *Eschericia coli* [34,12]. This has also been shown in a metagenomic study which revealed that microbial community adapts to QAC when continuously exposed. A recent review has shown increasing evidence for co resistance and cross resistance between QAC’s and other important antibiotics and disinfectants.

### Limitations of the study

The main limitation of the study was samples from only one hospital in Malaysia were studied. As very limited data on antibiotic resistance pattern was available, antiseptic and antibiotic resistance could not be compared.

## Conclusions

In conclusion, although the MIC of the QAC’s tested against MDRAB is much lower than the concentration used in the hospital, the high prevalence of *qacE* gene in the studied isolates warns the continuous monitoring of antiseptics use in the hospital.
